# The Effect of Self-Efficacy on Visual Discrimination Sensitivity

**DOI:** 10.1371/journal.pone.0109392

**Published:** 2014-10-08

**Authors:** George Zacharopoulos, Nicola Binetti, Vincent Walsh, Ryota Kanai

**Affiliations:** 1 Institute of Cognitive Neuroscience, School of Psychology, University College London, London, United Kingdom; 2 Sackler Centre for Consciousness Science, School of Psychology, University of Sussex, Sussex, United Kingdom; Birkbeck, University of London, United Kingdom

## Abstract

Can subjective belief about one's own perceptual competence change one's perception? To address this question, we investigated the influence of self-efficacy on sensory discrimination in two low-level visual tasks: contrast and orientation discrimination. We utilised a pre-post manipulation approach whereby two experimental groups (high and low self-efficacy) and a control group made objective perceptual judgments on the contrast or the orientation of the visual stimuli. High and low self-efficacy were induced by the provision of fake social-comparative performance feedback and fictional research findings. Subsequently, the post-manipulation phase was performed to assess changes in visual discrimination thresholds as a function of the self-efficacy manipulations. The results showed that the high self-efficacy group demonstrated greater improvement in visual discrimination sensitivity compared to both the low self-efficacy and control groups. These findings suggest that subjective beliefs about one's own perceptual competence can affect low-level visual processing.

## Introduction

Can subjective beliefs about one's own perceptual competence change one's perception? Traditionally, facilitation of low-level perceptual skills has been primarily attributed to two mechanisms: attention and visual perceptual learning. For example, previous psychophysical studies of vision showed that selective attention [1], [2] and feature-based attention [3], [4] can generate perceptual improvements. In addition, previous visual perceptual learning studies demonstrated perceptual improvements specific to the stimulus attributes used in training (e.g. orientation, spatial frequency, motion direction: [5], [6], [7]. However, whether simply having different subjective beliefs on one's own perceptual ability modulates perceptual performance remains to be determined.

Many psychological models of behavioral change have been proposed to explain and predict improvements in task performance to date. Self-efficacy (SE) theory [8], perhaps the most widely used model of behavioural change, has provided a novel social-cognitive account of how change in behavioral performance change occurs. The SE theory predicts that behavioural change is a direct function of the individual's beliefs in one's ability to exercise control over that particular behaviour [8]. Influences of SE on objective performance have been empirically demonstrated in a wide range of tasks including physical stamina [9], cognitive performance [10], and pain control [11], [12] amongst others. Given the ubiquitous effects of SE on performance, one might ask whether these effects generalise to low-level perceptual skills despite the fact that perceptual sensitivity is known to be a relatively stable trait within individuals that cannot be easily changed without prolonged training [13], [7].

In this study, we tested whether subjective beliefs of one's perceptual ability affect low-level visual discrimination sensitivity (VDS) in two visual tasks (contrast and orientation discrimination). In these tasks, participants made objective perceptual judgments on the contrast or the orientation of visual stimuli. After the completion of a first block of trials, we gave fake social-comparative feedback in order to manipulate participants' level of SE concerning their task performance. Participants assigned to the high SE groups were given positive feedback about their performance, that is was much better than average, whereas participants assigned to the low SE groups were given negative feedback about their performance, that it was worse than the average (see Methods Section for details). We hypothesized that for both the orientation and contrast task, participants assigned to the high SE groups would exhibit greater VDS improvements compared to participants in the low SE groups.

## Materials & Methods

### Participants

One-hundred and eighteen people (69 women) with normal or corrected-to-normal vision participated in this study, the majority of whom (n = 73) were university students. (The sample size per group can be seen in Table S1). This study, which was described to the participants as concerning contrast and orientation perceptual judgments, was advertised online to the University College London (UCL) Psychology subject pool database. Participants gave written and informed consent. The study was approved by the local ethics committee of University College London.

### Stimuli

In both tasks, the visual stimuli consisted of six sinusoidal vertical gratings (2.8 visual degrees in diameter, spatial frequency of 2.2 cycles per visual degree), which were radially arranged (eccentricity of 6.9 visual degrees) around a central fixation cross. The stimuli were presented on a calibrated CRT monitor (size 22″, spatial resolution of 1024×768 pixels, refresh rate of 60 Hz). The stimuli were presented in a darkened room with the computer monitor providing the only significant source of light. The stimuli, and the experimental procedure, were implemented in MATLAB (Mathworks Inc., Natick, MA, USA) using Psychtoolbox [14].

### Tasks and procedure

#### Visual discrimination tasks

**Figure 1:**
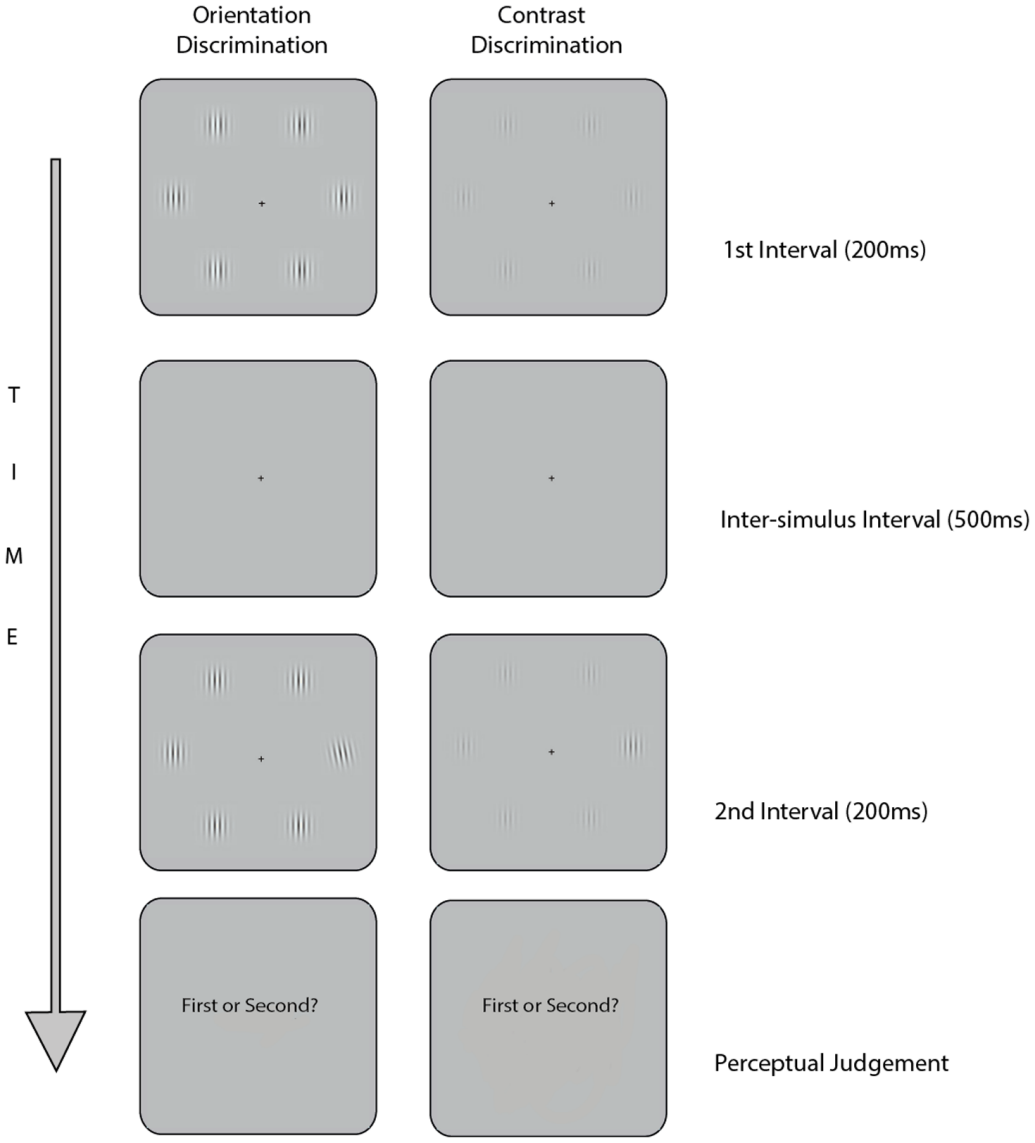
A schematic diagram of the experimental paradigm. Left: Contrast Discrimination task, Right: Orientation Discrimination task. Participants made a two-interval forced choice judgment on which temporal interval (i.e. first or second) contained the grating that popped out in contrast (left panel) or orientation (right panel).

We measured VDS in both contrast and orientation discrimination tasks (Figure 1). On a trial-by-trial basis, participants made objective perceptual judgments on the contrast (contrast task) or the orientation (orientation task) of the visual stimuli. In every trial, the visual stimuli were presented twice, with each presentation lasting 200 ms, and an inter-stimulus interval lasting 500 ms. In one of the two presentation intervals, the six vertical sinusoidal gratings were identical, while in the other interval, one of the six gratings (also named “*pop-out grating*”) differed from the rest either by a greater contrast (contrast task) or an oblique orientation (orientation task). The spatial position of the pop-out grating varied randomly between trials. Upon the presentation of the two intervals, participants were asked to choose, within 4 s, which of the two intervals contained the pop-out grating. The difficulty of the visual judgments, i.e., the parameter (measured in percentage in the contrast task and in degrees in the orientation task) of the pop-out grating, was varied using a 2-up-1-down staircase fashion [15]. Two consecutive correct visual judgments led to the parameter of the pop-out grating in the next trial being one step lower than in the previous trials, whereas one incorrect visual judgment led to an increase in the parameter of the pop-out grating. The step size of the parameter was.005% for the contrast task and 0.25 degree for the orientation task. Both tasks consisted of three blocks of trials termed “practice”, “pre-manipulation” and “post-manipulation”. In all three blocks, the starting parameter of the pop-out gratings was fixed in advance (i.e. Orientation: practice  = 10° pre-manipulation  = 5° post-manipulation  = 5°. Contrast: practice  = 60% pre-manipulation  = 45% post-manipulation  = 45%). Each block continued until the staircase completed fifty reversals, typically lasting around nine minutes. The threshold for each block was obtained by averaging the stimulus parameter of the pop-out grating over the final ten reversals.

#### Self-efficacy measurements

**Figure 2:**
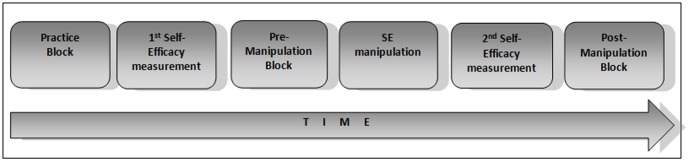
A schematic depiction of the experimental timeline as a function of time.

SE was assessed by questionnaire (Figure S1, S2). Each participant completed the SE questionnaire twice, once before the pre-manipulation block and once after the SE manipulation (Figure 2). Therefore, the score of the first questionnaire represented the baseline level of SE after the initial practice block, while the second questionnaire score reflected the SE level induced by manipulation with fake feedback.

Both questionnaires were constructed and administered using the standard methodology of SE scales [16], [17]. This included unipolar scales for items representing gradation of challenges phrased in terms of *can do* statements [16]. Both questionnaires were recorded on a 100-point scale, ranging in 10-unit intervals from 0–10 (complete uncertainty); through intermediate degrees of assurance, 50 (moderate certainty); to complete confidence, 90–100 (complete certainty). The aggregate of the question sub-scores divided by ten yielded the total SE score, which ranged from 10 (minimum SE) to 100 (maximum SE).

#### Experimental design

This study had two independent variables, VDS and SE. VDS had two levels representing two visual tasks (i.e. orientation and contrast) and three levels of SE (i.e. high, low and control). The effects of the experimental manipulation were assessed based on the measurements of the two dependent variables, SE and VDS. SE was measured as the difference in the SE questionnaire scores before and after the fake feedback manipulation, while VDS was measured as the change in VDS thresholds before and after the SE manipulation by fake feedback. Overall, the study consisted of six groups of participants: three groups representing each level of the SE (i.e. high, low and control) and the two tasks (i.e. orientation and contrast).

#### Experimental procedure

Each participant completed either the contrast or orientation task (Figure 2). Participants were seated 67 cm away from the monitor where they sequentially completed three blocks of visual task trials: practice, pre-manipulation and post-manipulation. The practice phase was needed to accommodate participants with the conditions of the experiment and to stabilize the participants' performance and minimize any immediate practice effects that could be found in the initial practice trials. After completing the first questionnaire, participants completed the pre-manipulation block at the end of which they were presented with a fake social-comparative feedback and fictional research findings (see below for details) about their performance. They were then again asked to complete the same SE questionnaire, for the next block (i.e. post-manipulation). The purpose of the experimental procedure was to assess how both SE and VDS were altered in response to the SE manipulation. After the completion of the experiment a brief interview took place to assess whether participants noticed the two deceiving aspects of the study (fake social-comparative feedback and the fictive research findings). The data from all seven participants who reported their concern about the accuracy of the feedback were excluded from the analyses.

#### Self-efficacy manipulation

To manipulate SE we utilized bogus normative comparison and conception of ability. The former was delivered by providing participants, upon the completion of the second block (i.e. pre-manipulation), with a two-digit number which was said to represent the percentile of participants' VDS performance; in relation to that of the other participants. To enable participants to understand who they were compared against, all subjects were informed that our sample was primarily composed of other undergraduate students and partly of adults from the general population. The participants assigned to the high SE and low SE groups were provided with the message (“Score: 81% percentile”) and (“Score: 39% percentile”) respectively. The participants in the control group were apprised that their performance percentile would appear at the end of the experiment. This was done to control for any potential social-comparative influence on SE or VDS.

Conception of ability was manipulated by giving participants fictional scientific information regarding the nature of the ability required for performing the experimental task successfully. Participants assigned to the high SE groups were apprised that there is a strong congruence among previous scientific studies that the ability for successfully performing the experimental task is entirely malleable, and that a small amount of practice is sufficient for enhancing this ability dramatically. Participants assigned in the low SE groups however, were informed that this ability does not change with practice. Control-condition participants were informed that previous research is ambiguous with regard to the extent to which the underlying ability is malleable or fixed. Apart from probing the effects of social-comparative influences, the reason for including control groups was to probe whether the mere completion of the questionnaires or the mere completion of the pre-manipulation phase (or SE questionnaires) had any impact on either of the two dependent variables. However, the addition of the control group was not central to the hypothesis concerning the present study, which was interested in the direct comparisons of the different levels of SE on VDS, but was mainly used as a reference point. As noted, it was added to assess whether the mere completion of the pre-manipulation block had any effects on the two measures (i.e. SE score and perceptual threshold) during the post-manipulation block (i.e. whether the two blocks interacted).

There are many ways to alter SE beliefs [16] but the two-fold approach utilised here is considered to be the standard method for both theoretical and empirical reasons. From the participants' perspective, the fake normative feedback constitutes their failure or success in the actual performance, which, according to the SE theory, constitutes the pivotal source of information influencing one's SE [8]. Regarding the fictive scientific narrative, if the performance on a task is fixed then there is no room for exerting personal mastery; therefore SE tends to deteriorate. If on the other hand the performance of a task is believed to be malleable, then one can exert a personal mastery which induces SE elevation [18], [19], [20], [16]. As for empirical supports, this type of self-efficacy induction has been shown to generate substantive alterations in self-efficacy beliefs across different tasks including pain control [12], problem solving [21], [10], acquisition of declarative knowledge [22], management skills [23] and complex decision making [18]. In short, both positive socio-comparative feedback and perceived malleability of a task has been shown to enhance while negative socio-comparative feedback or perceived non-malleability of a task has been shown to decrease self-efficacy beliefs.

While the two types of SE induction methods outlined here are conceptually different, we complement the socio-comparative feedback with the fictive scientific stories in order to maximise the changes in participants' SE beliefs (in either direction, namely increasing or decreasing self-efficacy).

#### Data Analysis

All statistical analyses that include independent samples t-tests, Cohen's effect size value, Pearson correlations, one-way analysis of variance (ANOVA) and 3*2 mixed-design, ANOVA were performed on the Software Package for Statistical Analysis (SPSS for Windows version 19.0), [24].

## Results

### Self-efficacy induction

**Figure 3:**
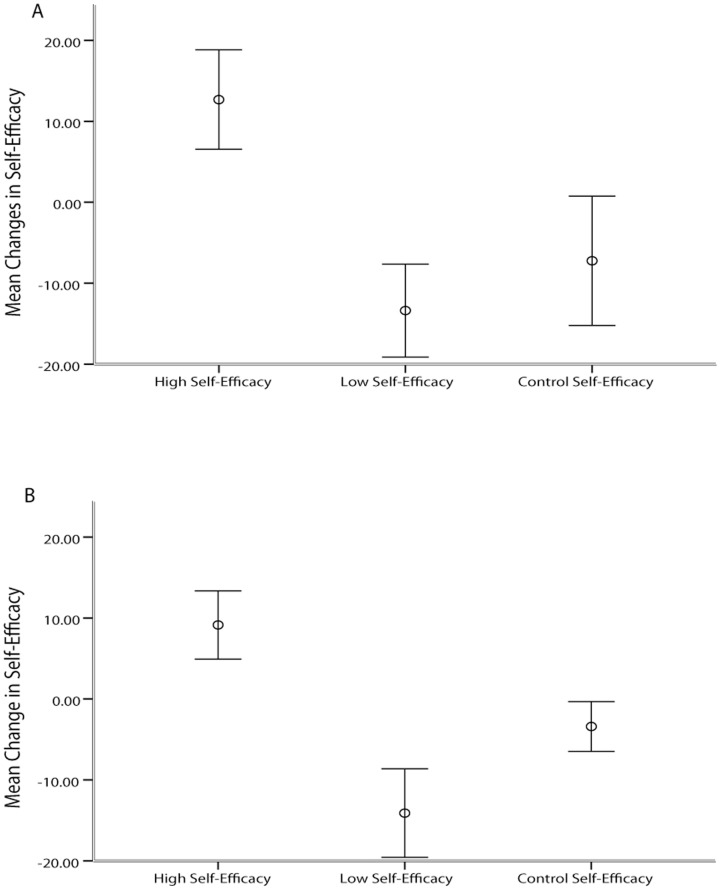
Magnitude of mean change in SE across groups in the A contrast and B orientation task. Error bars represent the confidence intervals surrounding the means (CI = 95%). For both tasks, participants assigned in the high SE group increased while participants assigned in the low SE group decreased their SE compared to the control group.

We first confirmed that our fake feedback manipulation successfully altered SE. Two independent-samples t-tests (for both contrast and orientation) were conducted to compare changes in SE between the positive and negative fake feedback groups. There was a significant difference in the changes in the SE scores for positive feedback groups (orientation, M = 9.14, SD = 9.25, contrast, M = 12.68, SD = 12.73) and the negative ones (orientation, M = −14.09, SD = 12.02, contrast, M = −13.36, SD = 11.89); orientation, t(40) = 7.018, p <.0005; d = 2.16, 95% CI [1.39, 2.92]; contrast, t(36) = 6.518, p <.0005; d = 2.11, 95% CI [1.30, 2.90]. (see figure 3A. contrast task 3B. orientation task).

We also conducted a 3*2 mixed-design analysis of variance with SE group as the between subject factor (i.e. high, low, control) and the pre-manipulation versus post manipulation phase as the within-subject factor was carried out on the changes in self-efficacy scores. We then performed planned contrast to compare the groups of interest. In the orientation task there was a significant interaction between group (i.e. high, low, control) and time (i.e. pre-manipulation, post-manipulation), F(2,59) = 30.929, p<.0005. Planned contrast revealed that high SE manipulation significantly elevated SE beliefs compared to the low SE manipulation, t(40) = −7.018, p<.0005. Similarly, in the contrast task, there was a significant interaction between group (i.e. high, low, control) and time (i.e. pre-manipulation, post-manipulation), F(2,53) = 18.904, p<.0005. Planned contrast revealed that high SE manipulation significantly elevated SE beliefs compared to the low SE manipulation, t(36) = −6.518, p<.0005.

To ensure that our groups did not differ initially we conducted two one-way between subjects ANOVAs to compare the SE scores between the three groups before the feedback manipulation (i.e. pre-manipulation). For the orientation task, there was no significant effect on SE scores for the three condition (F(2,59) = 1.036, p = .361). There was, however, a significant effect on SE scores for the three condition (F(2,53) = 3.227, p = .048) in the contrast task. Post-hoc comparisons of the three groups indicate that the high SE group (M = 54.32, SD = 9.62) gave significantly lower SE ratings than the low SE group (M = 64.21, SD = 12.58; p = .037). Searching for potential outliers (or delving into the individual scores) we identified four participants from the low SE group to have scored above 80/100 (particularly high) and 7 participants from the high SE group with a score below 45/100 (particularly low).

Therefore, as a response to the experimental manipulation in both tasks, participants assigned to the high SE group judged that they could perform the task more accurately compared to participants assigned to the low SE group. Taken together, these results confirm that the experimental manipulation successfully induced different levels of SE across groups as expected.

### Visual discrimination sensitivity

**Figure 4:**
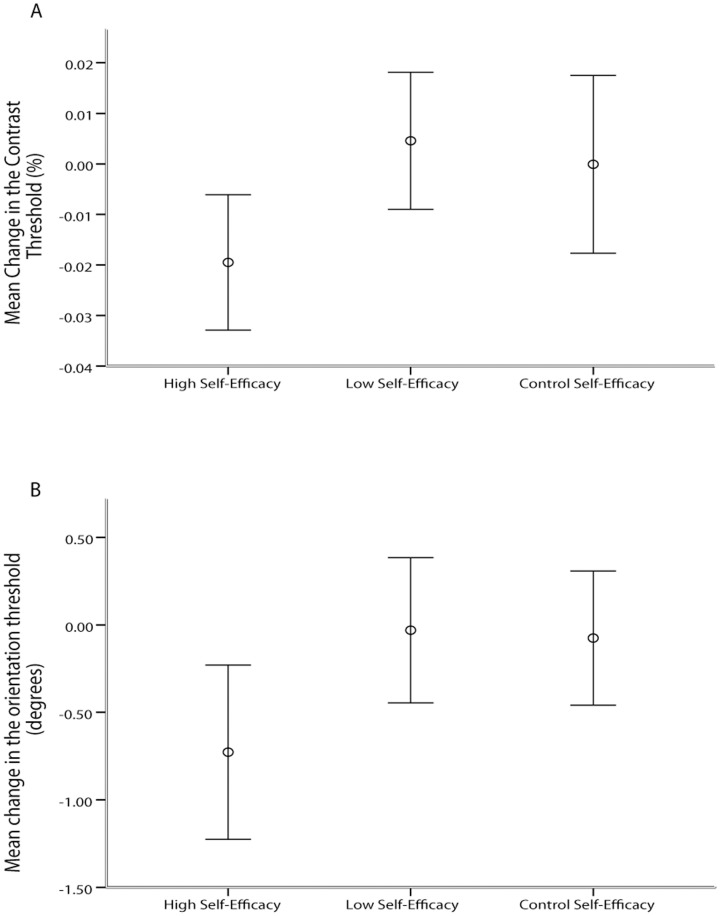
Magnitude of mean change in visual discrimination sensitivity across groups in the A contrast and B orientation task. Error bars represent the confidence intervals surrounding the means (CI = 95%). For both tasks, participants assigned in the high SE groups exhibited the greater increase in magnitude in VDS (decrease in discrimination threshold).

Having established the effectiveness of SE manipulation in our experiment, we next sought to test whether the experimental manipulation had a significant impact on objective VDS. We predicted that participants assigned in the high SE groups would exhibit greater improvements in their discrimination thresholds for the target grating compared to participants assigned in the low SE groups. Two independent-samples t-tests (orientation and contrast) were conducted to compare changes in VDS between the high and low SE groups. There was a significant difference in the changes in VDS between the positive feedback group (orientation, M = −.727, SD = 1.093, contrast, M = −.019, SD = .027) and the negative feedback group (orientation, M = −.043, SD = .909, contrast, M = .004, SD = .028); orientation, t(40) = −2.205, p = .033; d = −0.68, 95% CI [.04, 1.3]; contrast, t(36) = −2.652, p = 0.012; d = −0.86, 95% CI [.23, 1.51] (see figure 4A. contrast task 4B. orientation task; for individual VDS changes of all 118 participants, see figures S3, S4). These results indicate that experimental manipulation had successfully generated differential VDS across high and low SE groups as hypothesized.

As before, we also conducted 3*2 mixed-design analysis of variance with SE group as the between subject factor (i.e. high, low, control) and the pre-manipulation versus post manipulation phase as the within-subject factor was carried out on the changes in VDS. We then performed planned contrast to compare the groups of interest. In the orientation task there was a significant interaction between group (i.e. high, low, control) and time (i.e. pre-manipulation, post-manipulation), F(2,59) = 3.450, p = .038. Planned contrast revealed that high SE manipulation significantly elevated SE beliefs compared to the low SE manipulation, t(40) = −2.205, p = .033. Similarly, in the contrast task, there was a significant interaction between group (i.e. high, low, control) and time (i.e. pre-manipulation, post-manipulation), F(2,53) = 3.307, p = .044. Planned contrast revealed that high SE manipulation significantly elevated SE beliefs compared to the low SE manipulation, t(36) = 2.652, p = .012.

As before, to ensure that our groups did not differ initially we conducted two one-way between-subjects ANOVAs to compare the perceptual thresholds between the three groups before the feedback manipulation (i.e. pre-manipulation). No significant differences were found in the orientation (F(2,59) = .618, p = .542) or in the contrast task (F(2,53) = .426, p = .655).

Finally, the results of the control groups (i.e. without any SE manipulation) suggest that there was no consistent improvement or deterioration across participants in performance in the second block. Comparing the pre-manipulation and post manipulation scores using repeated measures t-test yields no significant results for either contrast (t(17) = .01, p = .992) or orientation (t(19) = .410, p = .687). The results indicate that pre- and post-manipulation blocks were independent and did not interact.

### Self-Efficacy as a moderator of visual discrimination sensitivity

**Figure 5:**
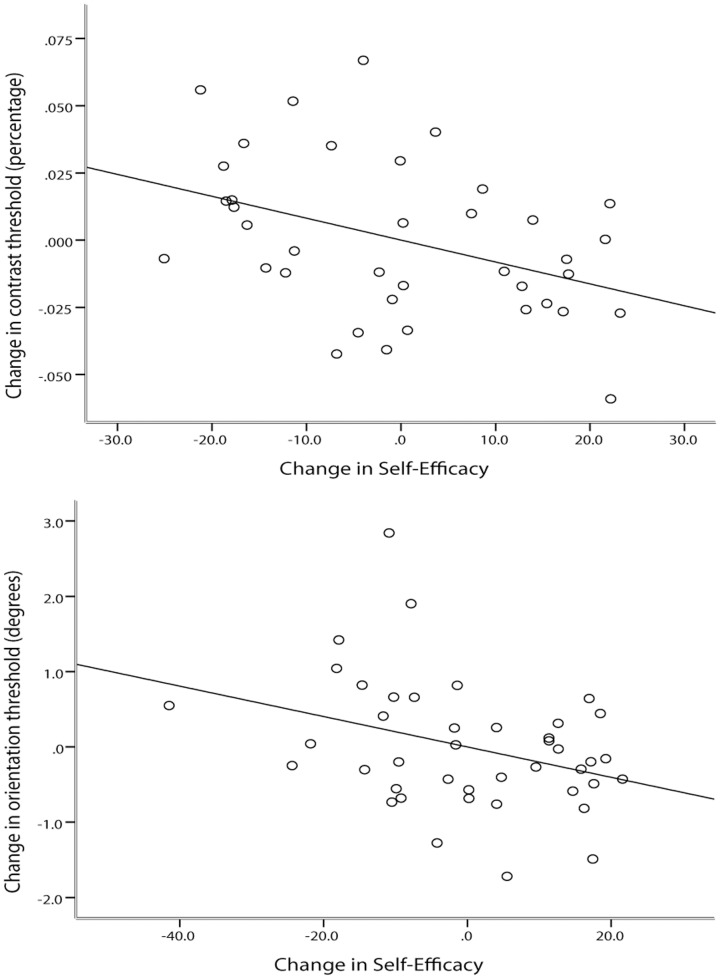
Scatter-plot of the two partial correlations performed on the A. contrast task and B. orientation task where change in SE was the predictor variable and change in VDS was the predicted variable in the. Each dot represents one participant. In both tasks SE moderately predict VDS.

To further establish the link between the changes in VDS and the induced changes in SE, we examined the partial correlation between them, using the experimental groups, while regressing out any individual differences in the objective VDS ability (i.e. past performance) and baseline SE. This approach for probing the mapping between SE and behaviour has been used in numerous studies (cited in Maddux, 1995). This analysis yielded a statistically significant correlation between the VDS change and the SE change both in the contrast (r = −.403, N = 38, p = .012, two-tailed) and in the orientation (r = −.342, N = 42, p = .027, two-tailed) tasks. These effects are graphically depicted in figure 5 (5A. contrast task, 5B. orientation task). This suggests that the greater the positive change in SE the greater the VDS improvement.

## Discussion

In this study we tested whether experimentally manipulating SE influences the performance of two low-level visual tasks: orientation and contrast discrimination. Firstly we found that the manipulation used was successful in altering SE as hypothesized and so participants assigned to the high SE groups increased, while participants assigned to the low SE groups decreased, their SE as measured by subjective questionnaire scores. Secondly we found that the experimental manipulation caused alterations in VDS. Specifically, participants in the high SE groups, compared to participants in the low SE groups, exhibited greater VDS improvements as shown by the corresponding threshold change (i.e. decreased threshold). Thirdly, correlation analyses showed that changes in the SE scores predicted changes in VDS. Specifically, the greater the positive magnitude of SE change, the greater the reductions of the pop-out grating threshold, thus the greater the VDS improvements.

These results are consistent with previous findings [9], [10], [11], [12] regarding the relationship between SE and behavioural performance. As was the case in the aforementioned previous studies, our results suggest that experimentally induced high SE significantly improves, while low SE does not significantly change, behavioural performance. However, the present study extends the previous studies of SE by providing the first evidence that high-level SE beliefs can reach and modulate low-level perceptual performances.

In addition, the current findings match those of previous studies showing that VDS is not a mere function of objective ability. Specifically, previous studies reported that VDS can be modulated by performance feedback [25], [26], [27]. Here we extended these findings by showing that subjective beliefs about one's visual discrimination ability modulates objective performance. Our results are also in agreement with a previous study by Shibata and colleagues (2009) showing that positive fake feedback indicating a larger performance improvement compared to negative feedback enhances VDS improvements. It is also worth mentioning that both in our present study and Shibata and colleague's study, negative feedback indicating a poor performance or smaller improvement had little effects on objective performance, possibly because subjects undermine negative feedback when it is much lower than expectation [28]; [29], [30]. A critical difference between this study and Shibata's study is that our fake feedback is based upon social comparisons with others, whereas their fake feedback was about the task performance per se. Nevertheless some overlapping mechanisms are likely to be at work in both cases. The relationships between different types of feedback remain to be determined in future work.

Various potential mechanisms might have generated the SE-induced performance improvements. In general, SE is thought to induce changes in behavioural performance through cognitive, affective or motivational processes [16]. Based on the experimental tools of the current study it is rather challenging to accurately discriminate between these three processes. However, given the nature of VDS there is a high likelihood that the mediating effect was of a rather motivational character. Our experimental design did not allow us to clearly separate the motivational factor. There are two potential ways to determine the contribution of motivation to our results in future studies. First, the degree to which these chain reaction possibilities hold true can be investigated by monitoring participants' changes in motivational state, via questionnaire administration, at certain time-points before and after the experimental manipulation. Simultaneous tracking of SE and motivation fluctuations would shed new light on their interplay. Furthermore, another approach would be to explicitly manipulate motivation independent of SE changes. This could be achieved in an experimental setting where participants in different SE groups (i.e. high, low, control) perform interesting and uninteresting VDS tasks. Measuring VDS performance by utilising such a double dissociation between SE and motivation would provide the stepping stone in elucidating the exact relationship between the two processes and their implication in affecting VDS.

Although not discussed in the SE literature, another possibility to be considered is that our experimental manipulation of SE may have affected the rate of perceptual learning mediated by neuronal plastic changes in early visual areas such as V1 [31], [32]. The involvement of perceptual learning could be assessed by examining the specificity of the effect of SE on VDS. For example, visual perceptual learning involving simple stimuli is known to be specific to the trained location [32]. As such, potential involvement of perceptual learning could be examined by testing whether effects of fake social comparative performance feedback generalize to untested locations or tasks. Observation of performance improvements at an untrained location would suggest that effects of SE on VDS involve different mechanisms than classic perceptual learning.

It is conceivable that the SE manipulation generated changes in participants' attentional or motivational levels. Namely, greater motivation may be induced by our positive feedback, and this may have in turn resulted in different levels of effort maintain attention to the task. With the current experimental design, we cannot exclude this possibility. On the contrary, motivation is an integral part of self-efficacy as it is known that self-efficacy influences motivation, leading to different levels of effort to complete a task [16]. It is thus highly probable that our SE manipulations influenced not just the subjective beliefs of self-efficacy, but also influenced the general motivational levels, and the differences found in the perceptual performance might be mediated by different levels of attentional engagement with the task.

Another important facet of the topic under investigation concerns the duration of the VDS (or even SE) changes. From this study alone we cannot determine how long the effects of SE manipulation lasted. However, previous studies, involving manipulation of SE and measurements of behaviour at different time-points revealed that the effects of SE-changes on behaviour might be long-lasting (smoking cessation: [33], obesity: [34]). It is difficult to make direct comparisons with our study since they differ in a number of important ways including the method of SE induction as well as the nature and in fact degree of complexity of the behaviours under investigation.

A potential limitation of the study involves the possibility that the efficacy-behaviour correlation might be a methodological artefact. Specifically, the documented results might reflect the participants' feelings of social pressure to match their performance to their SE ratings. To minimize such confounds, completion of SE scales occurred in complete privacy. In addition it has been demonstrated that participants are not particularly concerned about efficacy-behaviour matching and other studies [35] did not find evidence about any potential reactive effects of SE rating on subsequent behaviour.

Finally, our study offers novel aspects of metacognition in the realm of perceptual decision making. Metacognition of perceptual decision has been extensively studied in the context of visual awareness using retrospective confidence rating tasks [36], [37], [38], [39], [40]. However, the influence of prospective metacognitive judgments on subsequent perceptual performance has previously been neglected. Our experiments showed that prospective metacognitive judgments (i.e. SE) serve a self-fulfilling function. The magnitude of the SE score was primarily derived from the fake experimental manipulation information since it was a function of the group that participants were assign to, and the correlation between the changes in SE and changes in VDS shows a close link between subjective belief and objective performance. On the other hand, our participants must have experienced similar subjective feelings about the task difficulty during the pre-manipulation block, because the task difficulty was controlled by adaptive staircase for all participants. Thus, the information available for constructing the prospective metacognitive judgments is likely to be derived from one's subjective performance rather than actual performance. It remains to be investigated whether experimental alterations in SE (i.e. prospective metacognitive judgments) translate into overconfidence in the task and how that might influence retrospective metacognitive accuracy. Such research is likely to advance our understanding of how our subjective belief in our ability is interrelated with retrospective metacognitive judgements.

## Supporting Information

Figure S1 **Contrast Self-Efficacy Questionnaire.**
(TIFF)

Figure S2 **Orientation Self-Efficacy Questionnaire.**
(TIFF)

Figure S3 **Graphical depiction of the changes in VDS exhibited by all participants assigned to the contrast discrimination task, grouped per SE group (A: High, B: Control, C: Low).** The x axis represents all the participants comprising each group while the y axis represents the change in the pop-out grating threshold.
(TIFF)

Figure S4 **Graphical depiction of the changes in VDS exhibited by all participants assigned to the orientation discrimination task, grouped per SE group (A: High, B: Control, C: Low).** The x axis represents all the participants comprising each group while the y axis represents the change in the pop-out grating threshold.
(TIFF)

Table S1 **Sample size per each of the total 6 groups (118 participants).**
(TIFF)
