# Supplementary figures and images for: The Effect of Self-Efficacy on Visual Discrimination Sensitivity
Source: PLoS One. 2014 Oct 8;9(10):e109392. doi: 10.1371/journal.pone.0109392 (PMC4190082; doi:10.1371/journal.pone.0109392)

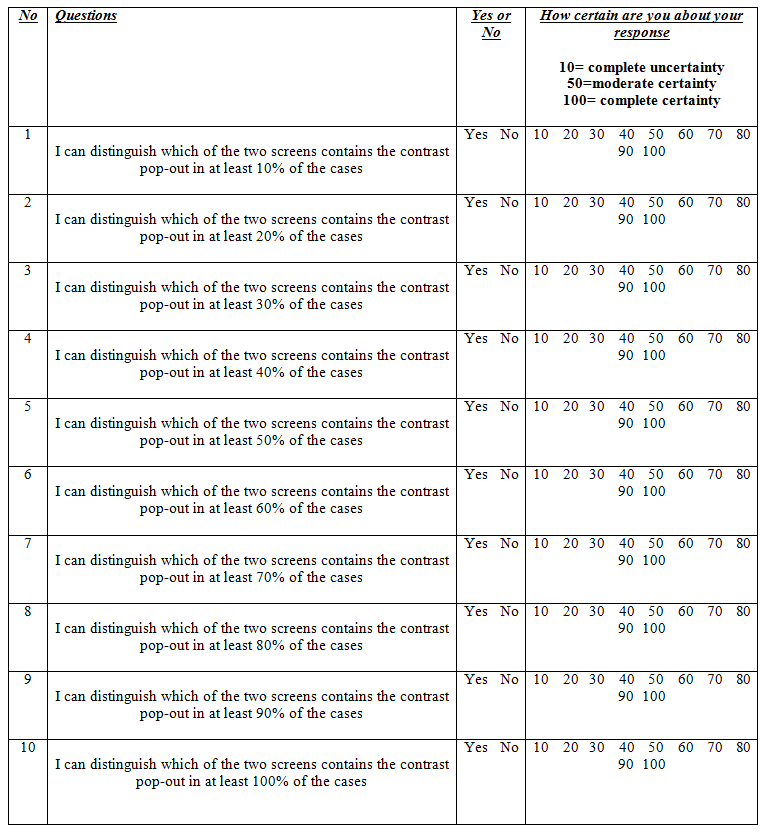

Supplement: Figure S1 — Contrast Self-Efficacy Questionnaire. (TIFF) [file pone.0109392.s001.tiff]

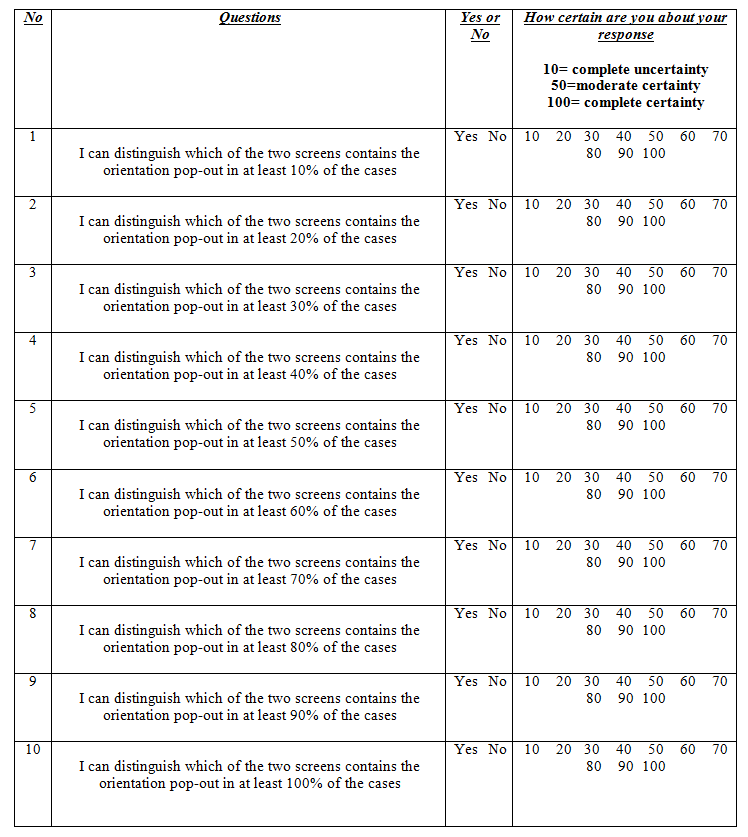

Supplement: Figure S2 — Orientation Self-Efficacy Questionnaire. (TIFF) [file pone.0109392.s002.tiff]

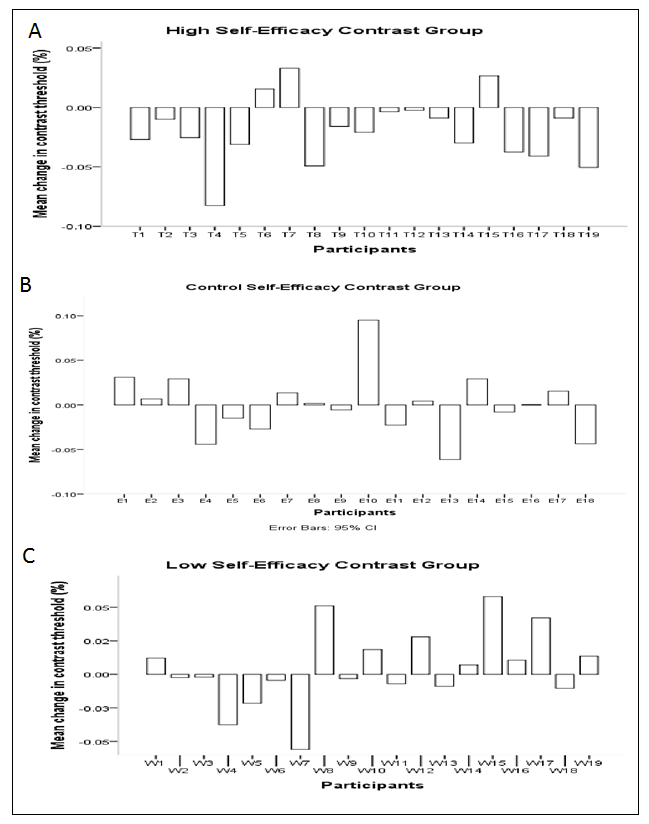

Supplement: Figure S3 — Graphical depiction of the changes in VDS exhibited by all participants assigned to the contrast discrimination task, grouped per SE group (A: High, B: Control, C: Low). The x axis represents all the participants comprising each group while the y axis represents the change in the pop-out grating threshold. (TIFF) [file pone.0109392.s003.tiff]

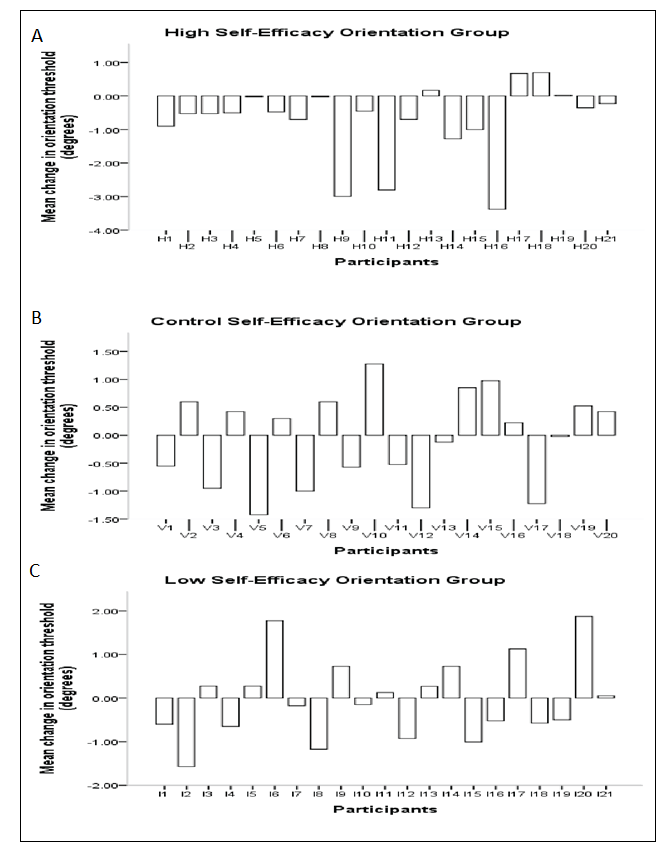

Supplement: Figure S4 — Graphical depiction of the changes in VDS exhibited by all participants assigned to the orientation discrimination task, grouped per SE group (A: High, B: Control, C: Low). The x axis represents all the participants comprising each group while the y axis represents the change in the pop-out grating threshold. (TIFF) [file pone.0109392.s004.tiff]

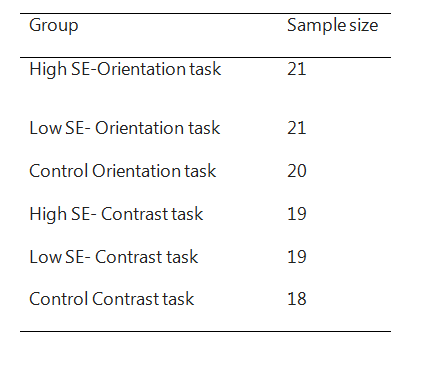

Supplement: Table S1 — Sample size per each of the total 6 groups (118 participants). (TIFF) [file pone.0109392.s005.tiff]
